# Effects of a novel microbial fermentation medium produced by *Tremella aurantialba* SCT-F3 on cigar filler leaf

**DOI:** 10.3389/fmicb.2023.1267916

**Published:** 2023-09-22

**Authors:** Qianying Zhang, Shuanghong Yang, Zhen Yang, Tianfei Zheng, Pinhe Li, Quanwei Zhou, Wen Cai, Yue Wang, Juan Zhang, Xiaoying Ji, Dongliang Li

**Affiliations:** ^1^Cigar Fermentation Technology Key Laboratory of China Tobacco (China Tobacco Sichuan Industrial Co., Ltd.), Cigar Technology Innovation Center of China Tobacco, Chengdu, China; ^2^Industrial Efficient Utilization of Domestic Cigar Tobacco Key Laboratory of Sichuan Province, Shifang, China; ^3^Science Center for Future Foods, Jiangnan University, Wuxi, China

**Keywords:** cigar filler leaf (CFL), *Tremella aurantialba*, fermentation medium, microbial community, volatile flavor compound

## Abstract

**Introduction:**

Adding a fermentation medium is an effective way to improve the quality of cigar tobacco leaves.

**Methods:**

A novel microbial fermentation medium produced by an edible medicinal fungus, *Tremella aurantialba* SCT-F3 (CGMCC No.23831) was used to improve the quality of cigar filler leaves (CFLs). Changes in sensory quality, chemical components, volatile flavor compounds (VFCs), and the structure and function of microbes were investigated during the fermentation process.

**Results:**

The sensory quality of CFLs supplemented with the *T. aurantialba* SCT-F3 fermentation medium significantly improved. Adding the fermentation medium increased the total alkaloid, reducing sugar, total sugar, and 12 VFCs significantly. A total of 31 microbial genera were significantly enriched, which increased the microbial community’s richness and diversity. Microbial functions increased, including nucleotide biosynthesis, amino acid biosynthesis, fatty acid and lipid biosynthesis, nicotine degradation, and nicotinate degradation. During fermentation, the total alkaloid, reducing sugar, and total sugar content decreased. The richness and diversity of the microbial community decreased, whereas bacterial enzyme activity increased. At the end of fermentation, the sensory quality was excellent. The microbial structure gradually stabilized, and functional genes were low. The contents of the four Maillard reaction products and three nicotine degradation products increased significantly. 2-Ethyl-6-methylpyrazine, methylpyrazine, D,L-anatabine, *β*-nicotyrine, nicotinic degradation products, and total nitrogen were significantly and positively correlated with sensory quality. Methylpyrazine, D,L-anatabine, and *β*-nicotyrine were negatively correlated with *Luteimonas*, *Mitochondria*, *Paracoccus*, *Stemphylium*, and *Stenotrophomonas*.

**Conclusion:**

This research provides not only a new microbial fermentation medium that utilizes edible and medicinal fungi to improve the quality of fermented CFLs, but also new ideas for the development and application of other edible medicinal fungi to improve the quality of cigar tobacco leaves.

## 1. Introduction

A cigar is a tobacco product made from fermented tobacco leaves and comprises three parts: a wrapper, binder, and filler. The wrapper determines the cigar’s appearance, and the binder fixes the position of the filler. As the most important component accounts for approximately 75% of the cigar’s weight, filler determines the style of the cigar. Fillers, rich in aroma and flavor, are essential for rolling superior-quality cigars. Fermentation is an important process to improve the quality of cigar filler leaves (CFLs). There are two fermentation methods for cigar tobacco leaves: spontaneous fermentation and artificial fermentation. When spontaneous fermentation using only microorganisms in the natural environment cannot meet the quality requirements of the product, fermentation media need to be artificially added to significantly improve the fermentation quality (China Tobacco Sichuan Industrial Co., Ltd., 2021). Traditional fermentation media include not only plant extracts, *Fritillaria cirrhosa* tincture, coffee tincture, cocoa tincture, holly gum, jujube juice, and cane sugar, but also fermented products, such as red rice (rice fermented by *Monascus*), sweet rice (rice fermented by *Rhizopus*), rice wine, and loquat wine (Jin, 1988; Cai et al., 2022; Hu et al., 2022; Zong et al., 2023). Novel fermentation media, strains such as *Bacillus cereus*, *B. pumilus*, *Acinetobacter*, and *Candida*, and enzymes such as proteases, amylases, cellulases, and pectinases, have been shown to improve the quality of CFLs (Li et al., 2012; Zhang et al., 2020a,b; Zheng et al., 2022a,b; Jia et al., 2023).

*Tremella aurantialba* SCT-F3 was a colloidal edible medicinal fungus isolated from *Naematelia aurantialba* (Bandoni & M. Zang) Millanes & Wedin. The fermentation broth of *T. aurantialba* was found to be rich in polysaccharides (Zhu et al., 2011; Deng et al., 2016; Sun et al., 2020), cellulose, hemicellulose, amylase, pectinase, protease, and lignin degradation enzymes (Yang et al., 2021). Polysaccharides are strong hydrophilic compounds with excellent moisture adsorption and retention capacities, which can improve tobacco’s moisture stability and ultimately improve tobacco production quality (Lin et al., 2020). In addition, the fermentation broth of *T. aurantialba* SCT-F3 has a complex aroma. It produces volatile aromatic components, including ethyl acetate, isobutanol, isoamylol, 2-methylbutan-1-ol, methyl isovalerate, furfural, methyl furan-3-carboxylate, 2-ethylhexanol, methyl benzoate, ethyl benzoate, methyl cinnamate, and globulol (Supplementary Table S1). We speculated that the fermentation broth of *T. aurantialba* may be a new fermentation medium that could improve the quality of ordinary CFLs.

In this study, we added the fermentation broth of *T. aurantialba* into CFLs to improve the quality. After fermentation, changes in sensory quality, chemical components, volatile flavor compounds (VFCs), and the structure and function of microbes were investigated, and the contributions of chemical components and VFCs to sensory quality and correlation analysis of the predominant microbes and VFCs were analyzed.

## 2. Materials and methods

### 2.1. Strain

*Tremella aurantialba* SCT-F3 (CGMCC No.23831) was isolated from *Naematelia aurantialba* (Bandoni & M. Zang) Millanes & Wedin and deposited at the China General Microbiological Culture Collection Center.

### 2.2. CFL fermentation

The fermentation medium was potato glucose broth containing 6 g/L potato extract powder and 20 g/L glucose and was autoclaved at 121°C for 15 min. *T. aurantialba* SCT-F3 was inoculated by sterile loops in 250-mL flasks with 75 mL fermentation medium and cultured at 150 rpm/28°C for 5 days. The fermented potato glucose broth was centrifuged (5,000 × g for 10 min) to remove mycelium pellets, and the supernatant was used as the fermentation medium. Unfermented potato glucose broth was used as the unfermented medium. Dexue No.1 from the original Chinese tobacco drying strips was used in this study. CFLs were loosened and spread flat on the table by hand. Mixed the medium and pure water in a ratio of 1:3 (v/v), then the mixture was evenly sprayed using an electronic sprayer on the surface of the leaves with 20% inoculation amount (v/g). After 2 h, the medium and water were absorbed entirely by the CFLs. The CFLs were transferred to an oak barrel for fermentation. The oak barrels were placed in the fermentation room, the temperature of the fermentation room was controlled at 37°C, the relative humidity of the fermentation room was controlled at 75%, and the fermentation was carried out for 40 days. CFLs added with *T. aurantialba* SCT-F3 fermented medium were sampled at 0, 20, and 40 days and marked as J0, J20, and J40; CFLs added with unfermented potato glucose broth fermented were sampled at 0, 20, and 40 days and marked as P0, P20, and P40; uninoculated CFLs were marked as Control. Samples were taken from the four corners and central position of the oak barrel, and the five positions of the CLTs were ground and mixed for testing. All samples were mixed well, transferred into sterile bags, and stored at −20°C until further analysis. All experiments were performed in triplicates.

### 2.3. Sensory evaluation

The fermented CFLs were stemmed and dried to 15 ± 2% moisture content. The samples were hand-rolled to a length of 110 mm and a circumference of 47 mm, then placed in a temperature humidity chamber at a temperature of 20 ± 2°C and humidity of 62 ± 3% to balance the samples’ moisture to 13 ± 1%. Five assessors from the staff trained by the China National Tobacco Quality Supervision & Test Center evaluated the sensory quality of all samples to recognize the aroma, smoke, aftertaste, combustibility, and comfort level. The samples were identified and scored according to 0–9 points, ranging from weak to strong.

### 2.4. Chemical components analysis

The chemical components of total alkaloids, total nitrogen, reducing sugars, and total sugars in the CFLs were determined according to the continuous flow (potassium thiocyanate) method of the Tobacco Industry Standard for the determination of total alkaloids (YC/T 468–2013), total nitrogen (YC/T 161–2002), and water-soluble sugar (YC/T 159–2019) (Hu et al., 2022).

### 2.5. VFC analysis

VFCs in CFLs were assayed using headspace solid-phase microextraction coupled with gas chromatography–mass spectrometry (HS-SPME-GC–MS) as previously described (Zheng et al., 2022a,b). A 50/30 μm DVB/CAR/PMDS fiber (Supelco Inc., United States) was used for aroma extraction. The CFLs were pulverized using a grinder, and the powder (1.5 g) was placed in a 10–mL glass vial. The sample was extracted at 60°C for 30 min, and the fiber was inserted into the injection port of the GC device at 250°C for 1 min to desorb the analytes. The sample was analyzed on a DB-5MS column (60 m × 0.25 mm × 0.25 μm, Agilent Technologies, United States). The GC oven temperature was maintained at 40°C for 2 min, followed by an increase of 10°C/min to 250°C and then held for 5 min. Helium (purity: 99.999%) was used as the carrier gas at a constant flow of 1 mL/min. The mass spectrometer conditions were: EI voltage, 70 eV; ion source temperature, 300°C; mass range, m/z 35–350. The identification of volatile compounds in the extracts was based on a comparison of the mass spectra and retention times of the individual compounds with those of standard compounds deposited in the National Institute of Standards and Technology database in the MS device (SI and RSI ≥800). Peak areas were compared with those of the internal standard for quantification.

### 2.6. Microbial community analysis

Samples (5.0 g) were suspended in 250 mL sterile phosphate buffer saline and shaken for 2 h at 200 rpm, after which the supernatant was centrifuged (10,000 × g for 30 min). The total genomic DNA of each sample was extracted using an EZNA^®^ Soil DNA Kit (Omega, United States) according to the manufacturer’s instructions. The bacterial genomic DNA were amplified and sequenced using primers 515F and 907R for 16S rRNA genes (Parada et al., 2016). The fungal genomic DNA was amplified using primers ITS1F and ITS2R for internal transcribed spacer (ITS) genes (Usyk et al., 2017). Amplicons were pooled in equal amounts and sequenced using a 2 × 300 paired-end configuration and an Illumina MiSeq sequencing system (Illumina, United States). Operational taxonomic units (OTUs) of qualified sequences were identified using the clustering program VSEARCH version 1.9.6 and the SILVA version 132 database (Rognes et al., 2016) with 97% similarity. Alpha diversity, including the Chao1, Shannon, and Simpon values, was analyzed using QIIME version 1.9.1 (Caporaso et al., 2010). Phylogenetic Investigation of Communities by Reconstruction of Unobserved States (PICRUSt2) and Fungi Functional Guild (FUNGuild) were used to predict metagenomic functions based on normalized OTU tables (Douglas et al., 2019; Xie et al., 2021).

### 2.7. Statistical analysis

All experiments were repeated at least thrice. SPSS version 19 software (SPSS Inc., United States) was used to carry out a one-way analysis of variance and Duncan’s multiple comparison test (*p* < 0.05). Heat maps and cluster analyses were performed using R version 4.0.0. Galaxy 1 was used for the linear discriminant analysis of effect size (LEfSe) to assess significant differences in CFLs with different treatments. SIMCA-P version 13.0 software (Umetrics, Sweden) enabled a partial least-squares (PLS) analysis of the contributions of conventional chemical constituents and VFCs to the sensory evaluation. Additionally, to determine the correlation between representative microbes and core VFCs based on Spearman’s correlation coefficients (*p < 0.05*), network analysis was performed using the Gephi software.

## 3. Results

### 3.1. Results of sensory evaluation

**Figure 1:**
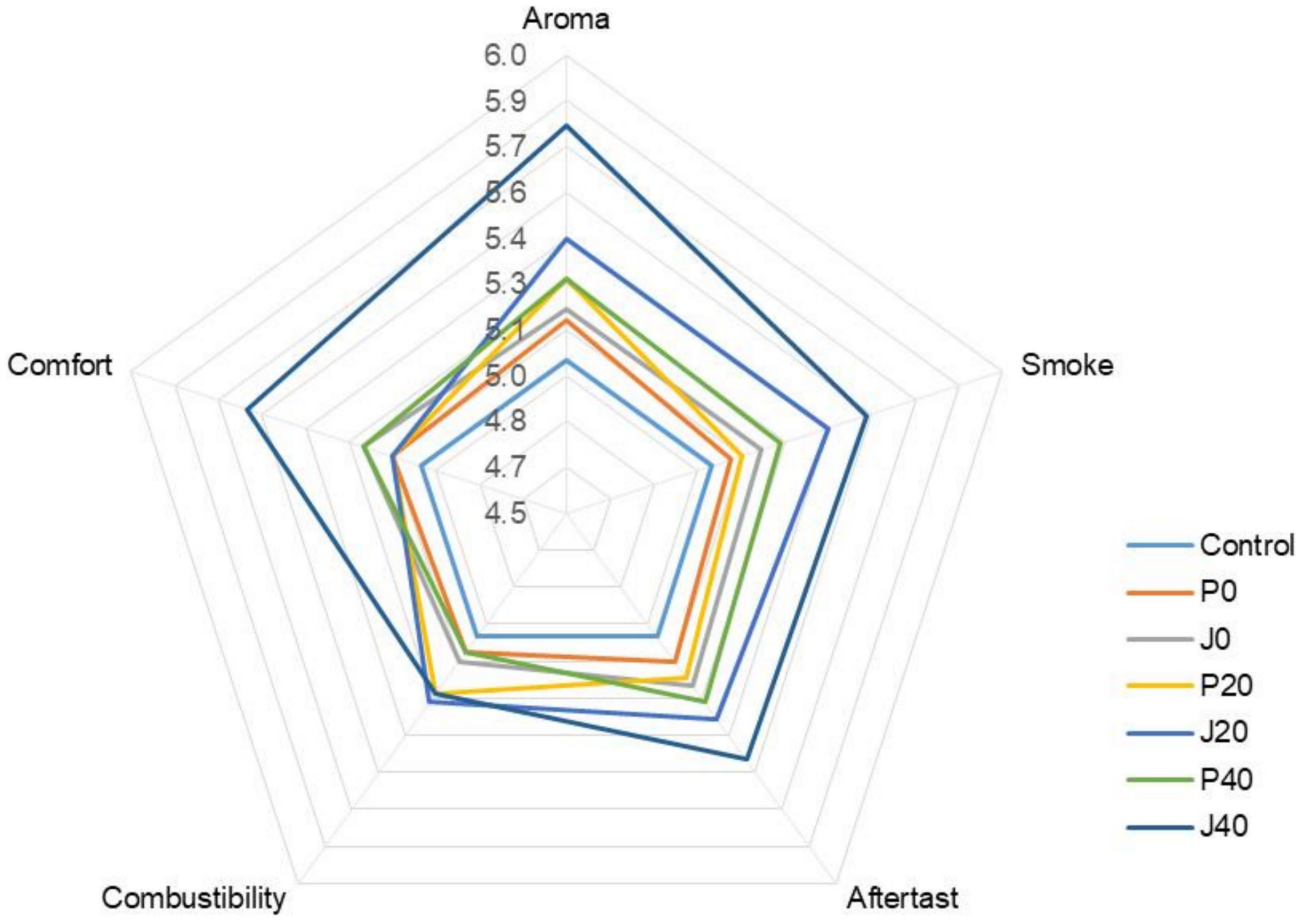
Plot of the sensory score of cigar filler leaves (CFLs). J0, J20, and J40 denote CFLs added with *T. aurantialba* SCT-F3 fermented medium at 0, 20, and 40 days; P0, P20, and P40 denote CFLs added with unfermented potato glucose broth fermented at 0, 20, and 40 days; Control denotes uninoculated CFLs.

Seven CFLs were evaluated and scored according to the cigar evaluation criteria. The detailed evaluation scores for each sample are shown in Figure 1. The scores of the fermented CFLs were higher than those of the control, and as fermentation progressed, the quality of the CFLs improved gradually. Except for combustibility, other qualities were significantly improved (*p < 0.05*) in those with *T. aurantialba* SCT-F3 fermentation medium (J0, J20, and J40) compared to those in potato glucose broth (P0, P20, and P40). Specifically, aroma mellow, richness, maturity, and sweetness increased; irritation decreased, and balance sense improved.

### 3.2. Profiles of chemical component

**Figure 2:**
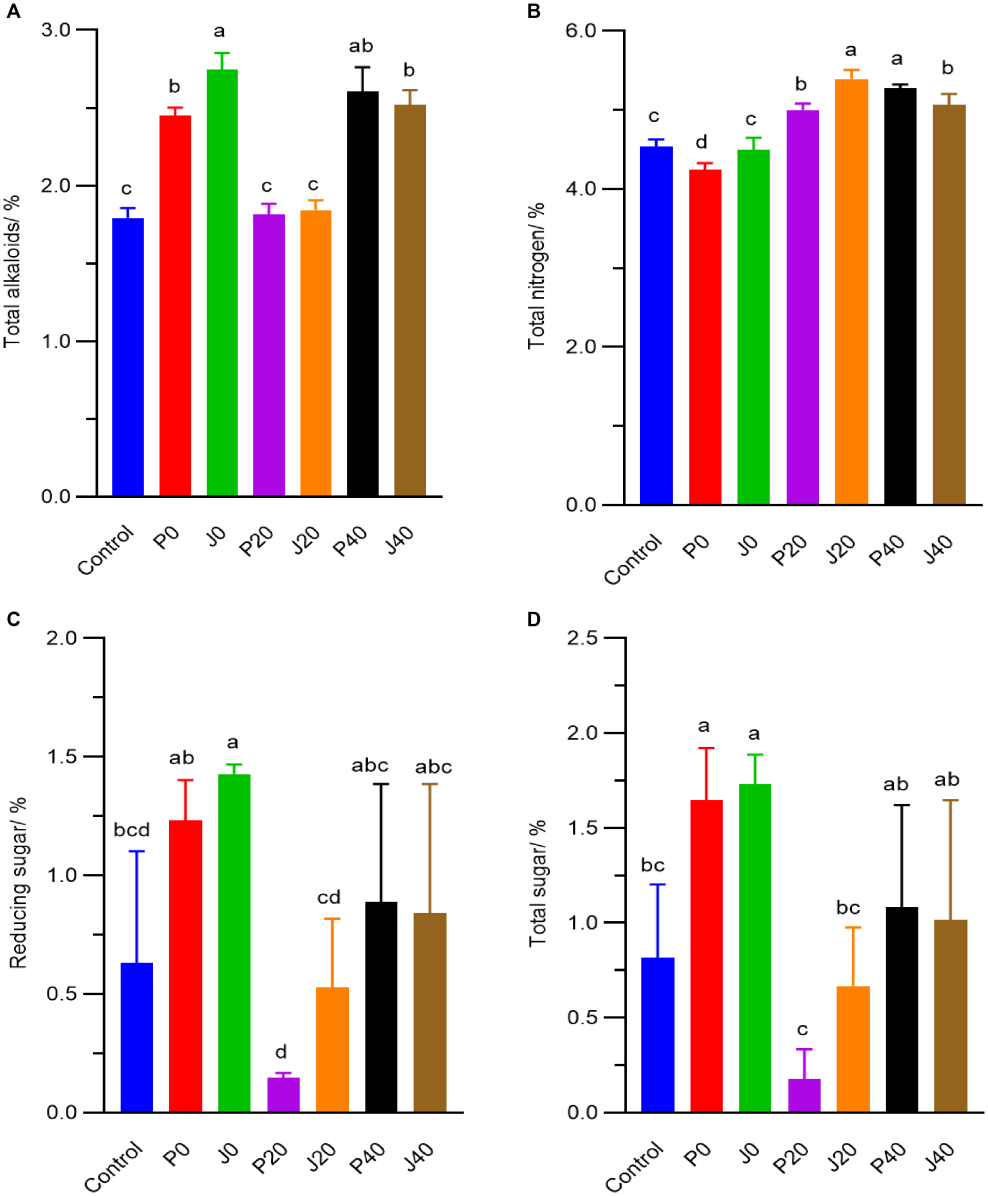
Contents of **(A)** total alkaloids, **(B)** total nitrogen, **(C)** reducing sugar, **(D)** total sugar of CFLs.

During fermentation, the contents of total alkaloid, total nitrogen, total sugar and reducing sugar fluctuated from 1.8–2.8%, 4.3–5.4%, 0.2–1.7% and 0.2–1.4%, respectively. The total alkaloid, reducing sugar, and total sugar contents significantly increased (*p < 0.05*) in samples supplemented with fermentation media on day 0 (F0 and P0), as shown in Figure 2. They then decreased to their lowest levels on day 20 (J20 and P20) and gradually increased (J40 and P40). The total nitrogen content at 20 and 40 days (J20, P20, J40, and P40) was higher than that on day 0 (J0 and P0) and in control.

### 3.3. Profiles of VFCs

**Figure 3:**
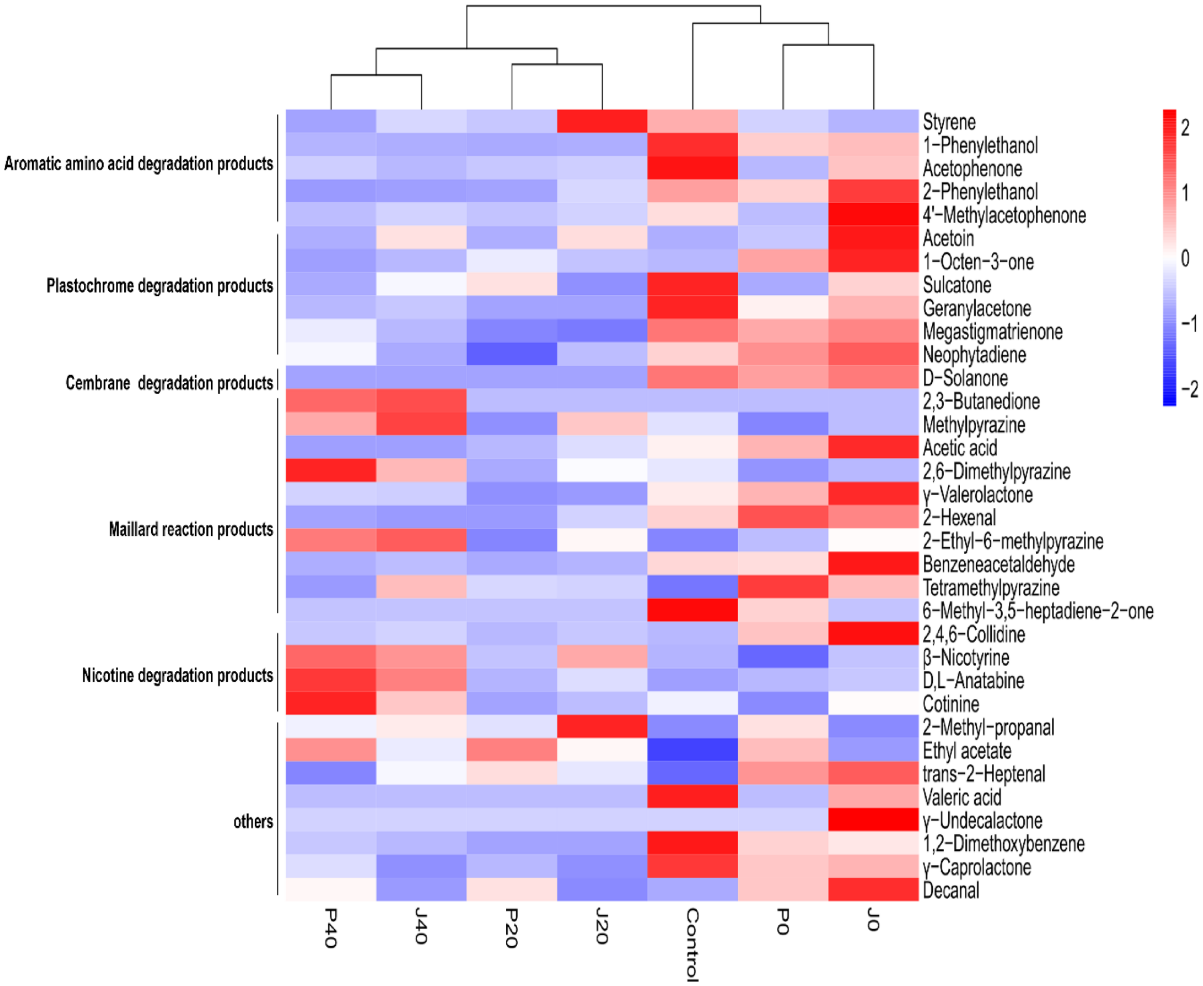
Hierarchical clustering of volatile flavor compounds in CFLs.

In total, 188 volatile components were detected in the CFLs, including 32 ketones, 20 aldehydes, 11 alcohols, and 10 esters. Furthermore, 34 VFCs (Figure 3), five aromatic amino acid degradation products, six plastochrome degradation products, one cembrane degradation product, 10 Maillard reaction products, and four nicotine degradation products were selected for further analysis. These compounds have been reported to have different aromas that may play important roles in the aroma profiles of cigars. The contents of aromatic amino acid degradation products, plastochrome degradation products, and cembrane degradation product, decreases gradually with fermentation, whereas four Maillard reaction products and 3 nicotine degradation products increased.

Cluster analysis divided the unfermented (control, P0, and J0) and fermented (J20, P20, J40, and P40) CFLs into two clusters, and the fermented CFLs were subdivided into two groups: P20 and J20, P40 and J40. The addition of fermentation medium caused a decrease in eight VFCs and an increase in 14 VFCs. Compared to the control, the 12 components of J0 increased. Styrene and 2-methyl-propanal levels peaked at J20. The vast majority of the VFCs changed after fermentation. Compared to the control, 15 VFCs in J40 decreased, 13 VFCs increased, 18 VFCs in P40 decreased, and 11 VFCs increased. Specifically, 2,3-butanedione, methylpyrazine, 2,6-dimethylpyrazine, 2-ethyl-6-methylpyrazine, *β*-nicotyrine, D,L-anatabine, cotinine, and ethyl acetate were enriched on day 40.

### 3.4. Contributions of chemical components and VFCs to sensory

**Figure 4:**
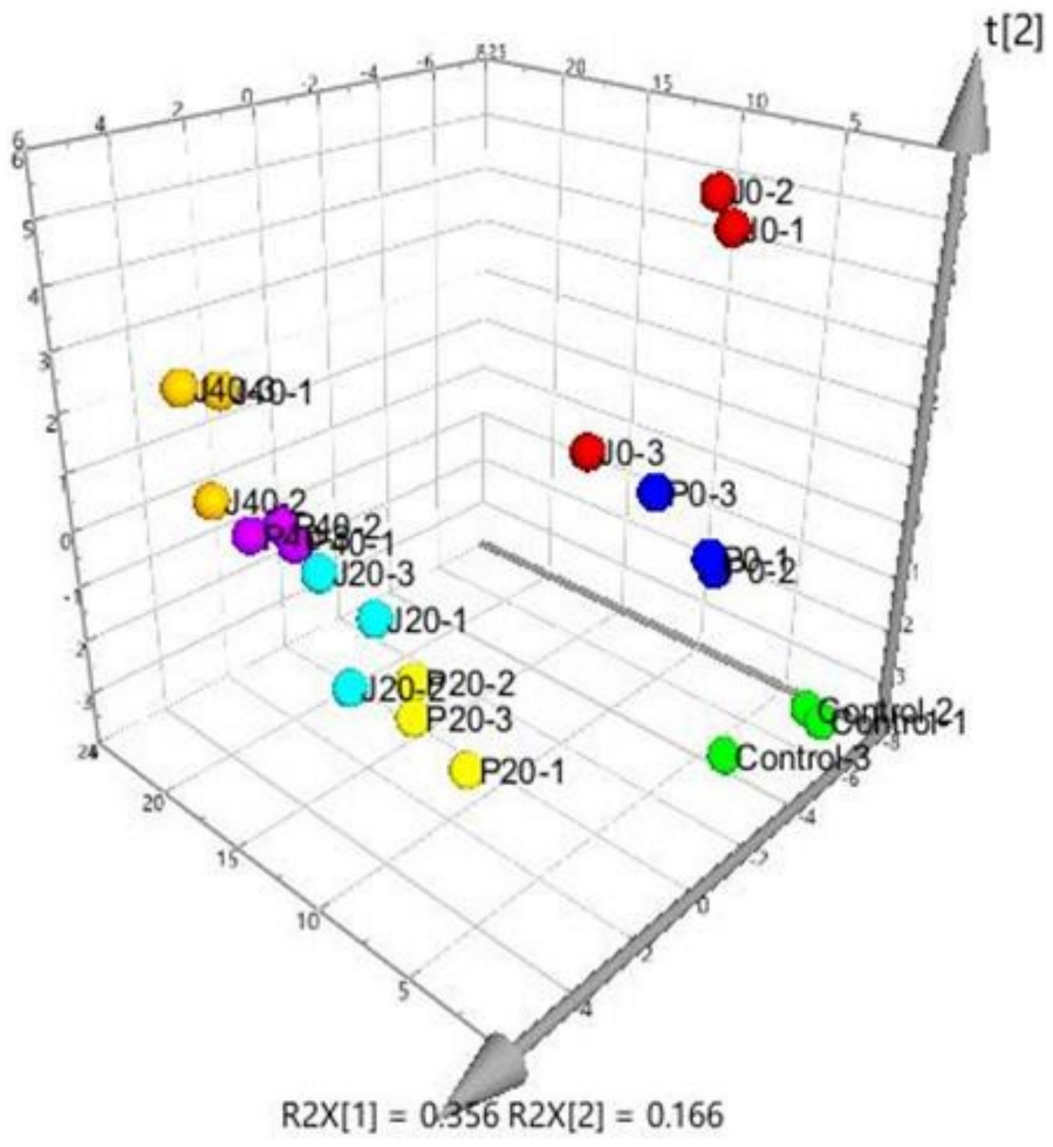
Score scatter three-dimensional plots of the partial least-squares regression (PLS). PLS of various CFLs is represented as a two-dimensional representation of the scores (t[1] and t[2]) on the first and second PLS components.

PLS was performed on scaled values of chemical constituents and VFCs as X-variables and on scaled values of total sensory evaluation scores as Y-variables for correlations. Two significant principal components of the total variance in the data matrix were extracted. The R2X (*cum*), R2Y (*cum*), and Q2 (*cum*) were 0.522, 0.808, and 0.701, respectively, which meant that 52.2% variation was due to these two components, with a total of 80.8% dummy Y-variables per class and 70.1% overall cross-validated R^2^ for these two components. The data indicated that the PLS model was suitable for this study. The distributions of the samples in the first and second components of the statistical analysis are shown in Figure 4. Groups J0 and P0 are located on the right side of the plot, J20 and P20 on the lower left, and J40 and P40 on the upper left. Table 1 shows that 14 variables with a VIP higher than 1.0 were important in the first and second most significant principal components. 2-ethyl-6-methylpyrazine, methylpyrazine, D,L-anatabine, *β*-nicotyrine, nicotinic degradation products, and total nitrogen were significantly positively correlated with the total sensory evaluation scores. In contrast, 1-phenylethanol, geranylacetone, megastigmatonone, D-solanone, 6-methyl-3,5-heptadiene-2-one, Maillard reaction products, 1,2-dimethoxybenzene, and *γ*-caprolactone were significantly negatively correlated with the total sensory evaluation scores.

**Table 1:** VIP values and correlation coefficients between variables and total score of sensory evaluation^†^.

Variables	VIP[1]	Coefficient values[1]	VIP[2]	Coefficient values[1]
1-Phenylethanol	1.61859	−0.04863	1.44917	−0.06656
Geranylacetone	1.37830	−0.04141	1.23103	−0.05064
Megastigmatrienone	1.26837	−0.03810	1.13300	−0.04933
D-Solanone	1.59346	−0.04787	1.42907	−0.05300
2-Ethyl-6-methylpyrazine	1.20992	0.03635	1.25876	0.07988
6-Methyl-3,5-heptadiene-2-one	1.59149	−0.04781	1.54943	−0.09273
Methylpyrazine	1.48244	0.04454	1.47742	0.09068
D,L-Anatabine	1.19771	0.03598	1.08241	0.05396
*β*-Nicotyrine	1.31881	0.03962	1.18420	0.05628
1,2-Dimethoxybenzene	1.64664	−0.04947	1.50381	−0.07877
*γ*-Caprolactone	1.41570	−0.04253	1.26546	−0.05635
Maillard reaction products	1.46849	−0.04412	1.34514	−0.03941
Nicotinic degradation products	1.34320	0.04035	1.20795	0.05820
Total nitrogen	1.71488	0.05152	1.54539	0.07565

^†^Means significance at the level of 0.05. The coefficient is significant (*p* < 0.05). VIP[1] and VIP[2] represent the VIP of the first and second principal components, respectively, and the coefficient values [1] and [2] represent the coefficient values of the first and second principal components, respectively.

### 3.5. Overview of microbial community

**Figure 5:**
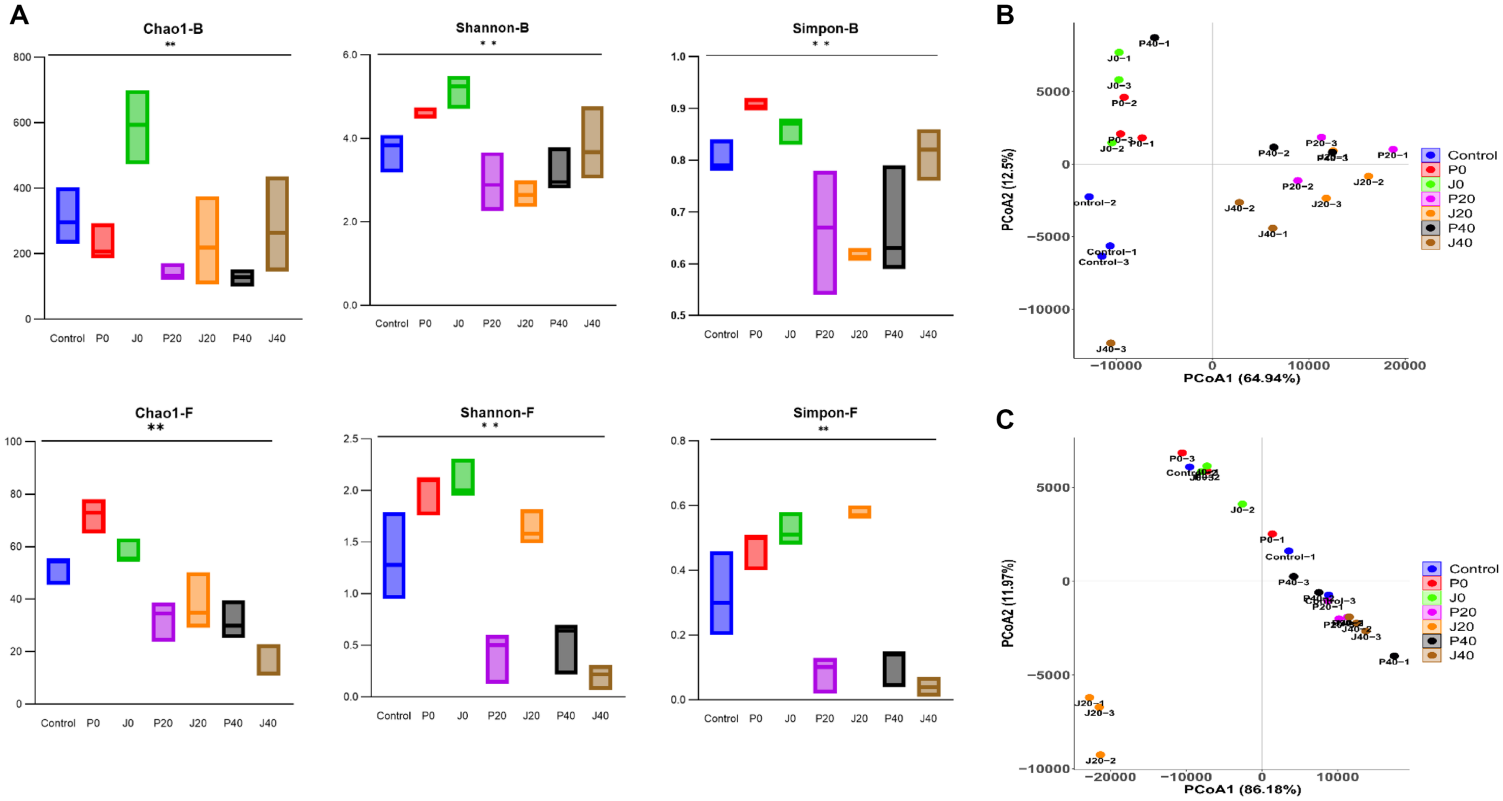
Diversity of microbial communities in CFLs. Alpha diversity was determined based on the Chao1, Shannon, and Simpson indices **(A)**. Bacterial beta diversity **(B)** and fungal beta diversity **(C)** were measured by weighted UniFrac distance. ^**^means significance at the level of 0.01.

Figure 5A shows alpha diversity indices, including the Chao1, Shannon, and Simpson indices, of bacterial and fungal communities in CFLs. The Chao1, Shannon, and Simpson values of the bacterial communities were higher than those of the fungal communities, indicating that the richness and diversity of the bacterial communities were generally higher than those of the fungal communities. The Chao1 values of J0 were higher than those of others, indicating that the richness of the bacterial and fungal communities increased after adding the *T. aurantialba* SCT-F3 fermentation medium. The Shannon and Simpson values of microbes in CFLs with medium without fermentation (P0 and J0) were higher than those in the other samples, indicating that adding fermentation medium increased the diversity of the microbial community. As fermentation progressed, fungal alpha diversity indices decreased.

Unconstrained principal coordinate analysis of weighted UniFrac distance revealed that the microbiota of CFLs with different treatments explained 77.4% of the bacterial variance (Figure 5B) and 98.1% of the fungal variance (Figure 5C). The addition of the fermentation medium altered the structure of the bacterial and fungal communities. The fungal community structure of sample J20 significantly differed from the other samples.

### 3.6. Changes in the structure of microbial flora

**Figure 6:**
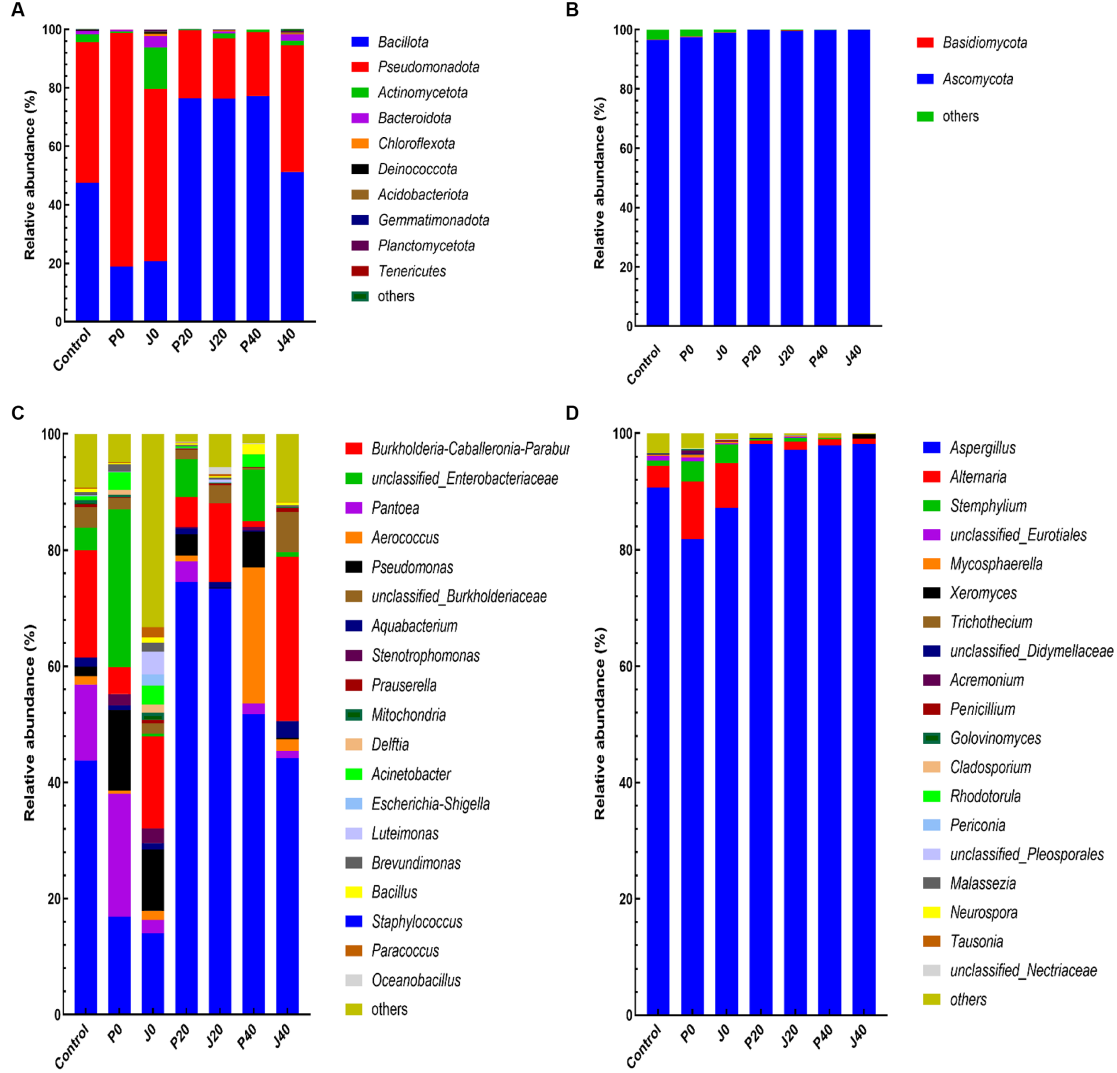
Plot of the phylum and genus level relative abundances for the bacterial and fungal communities in CFLs. Bacterial communities at the phylum **(A)** and genus levels **(C)**, and fungal communities at the phylum **(B)** and genus levels **(D)**.

The abundance of bacterial and fungal taxa is shown in Figure 6. The dominant bacterial phyla were *Bacillota* and *Pseudomonadota*, and the dominant fungal phyla were *Ascomycota* and *Basidiomycota* (Figures 6A,B). *Pseudomonadota* and *Ascomycota* of unfermented samples (control, P0, and J0) were significantly higher than those of the fermented samples. The dominant bacterial genera, with relative abundances higher than 5.0% in at least one sample, were *Staphylococcus*, *Burkholderia-Caballeronia-Paraburkholderia*, unclassified *Enterobacteriaceae, Pantoea*, *Aerococcus*, *Pseudomona*s, and unclassified *Burkholderiaceae*, which represented between 44.6 and 96.1% of the total abundance in each CFL sample (Figure 6C). The relative abundance of *Staphylococcus* decreased significantly at P0 and J0. The dominant fungal genera were *Aspergillus* and *Alternari* (Figure 6D). The relative abundance of *Aspergillus* and *Alternari* significantly decreased in P0 and J0, whereas the relative abundance of *Stemphylium* showed the opposite trend.

**Figure 7:**
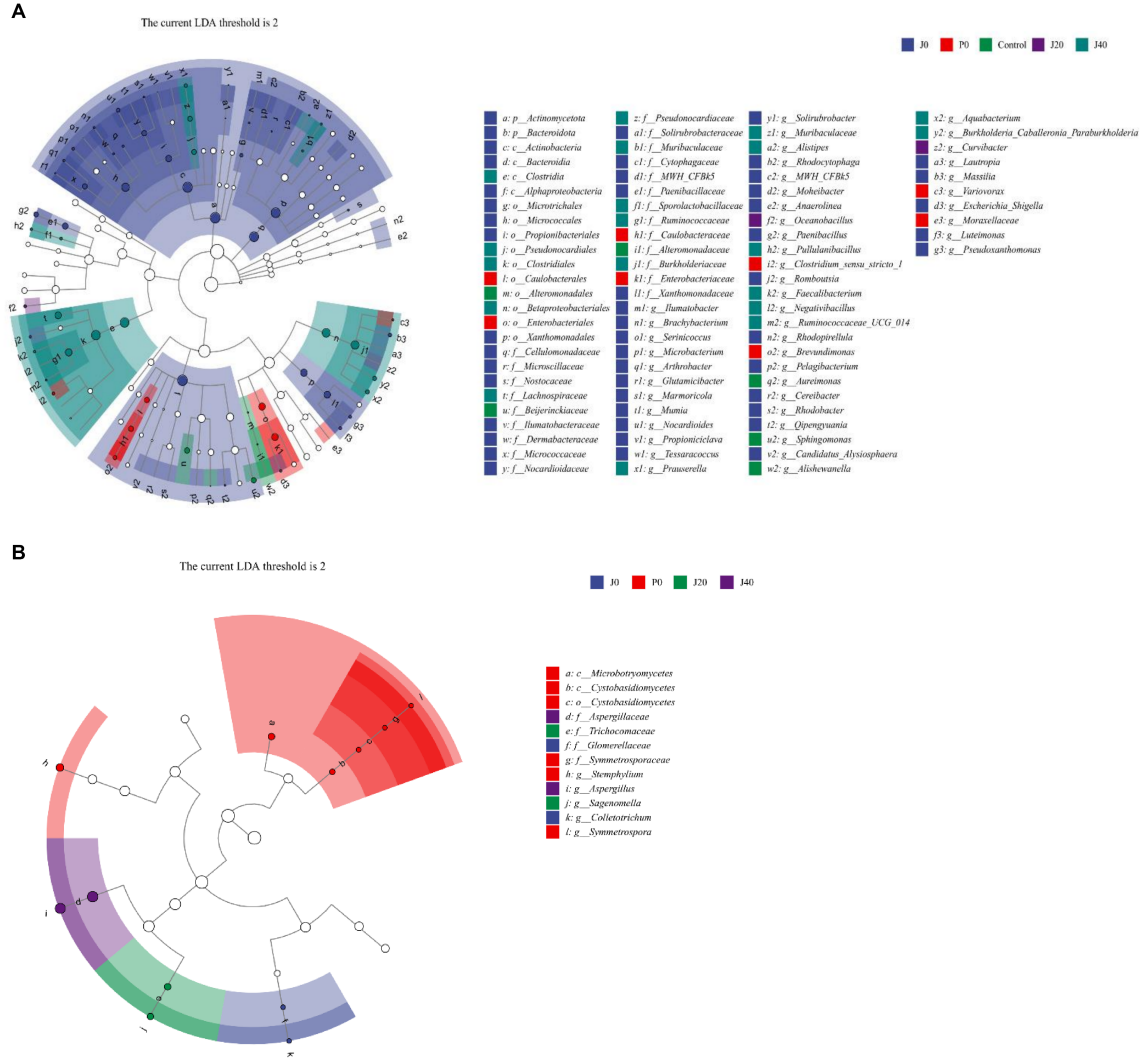
Evolutionary branch map of the bacteria **(A)** and fungi **(B)** significantly differs in CFLs.

LEfSe analyses revealed significant differences below the phylum level to explore the different microbes among the CFLs under different treatments (Figure 7). The circles from innermost to the outermost represent bacterial and fungal classifications from the phylum to genus levels, and the corresponding colors in every group denote bacterial and fungal taxa with a significant difference. Notably, 85 different bacteria appeared at the LDA threshold of 2, judging by statistically significant differences (*p < 0.05*), which consisted of two phyla, four classes, 10 orders, 22 families, and 47 genera (Figure 7A). In detail, 30 genera were significantly enriched in inoculated *T. aurantialba* SCT-F3 CFLs without fermented (J0), such as *Escherichia-Shigella*, *Luteimonas*, and *Brachybacterium*. Four genera were significantly enriched in P0: *Brevundimonas*, *Clostridium sensu stricto*1, *Variovorax*, and *Moraxellaaceae*. Two genera were significantly enriched in J20, including *Oceanobacillus* and *Curvibacter*. Eight genera were significantly enriched in J40, such as *Aquabacterium*, *Burkholderia-Caballeronia-Paraburkholderia*, and *Prauserella*. There were 12 fungi of two classes, one order, four families, and five genera (Figure 7B). *Colletotrichum* and *Symmetrospora* were significantly enriched in J0 and P0, *Sagenomella* was enriched in J20, and *Aspergillus* in J40.

### 3.7. Changes in microbial metabolic pathways

**Figure 8:**
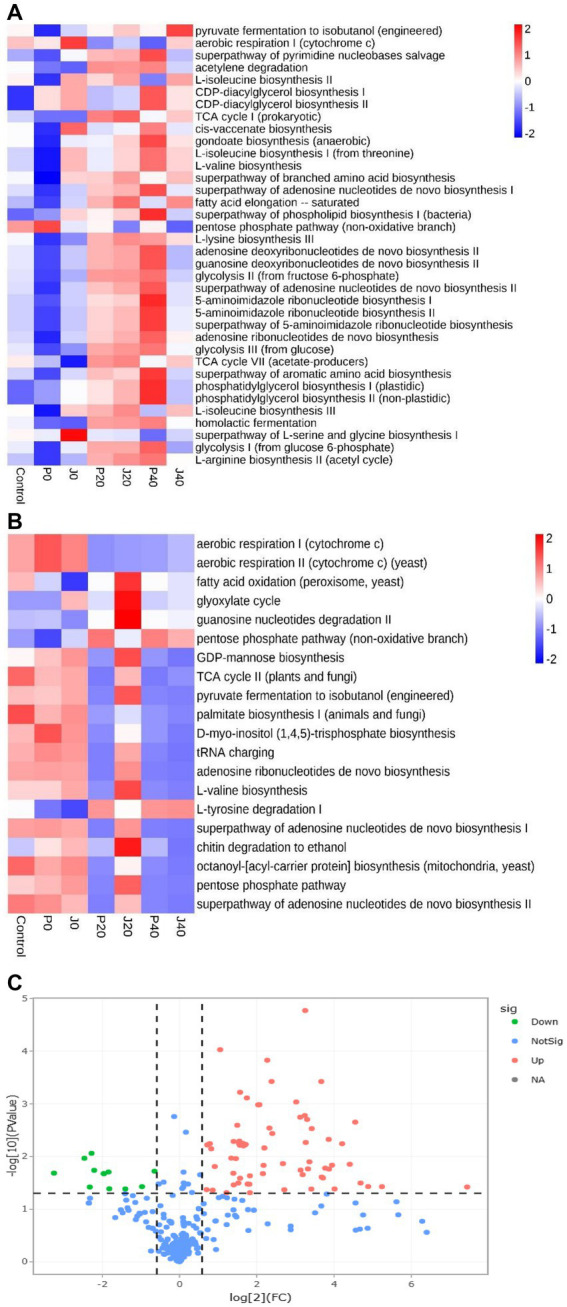
The relative abundance of bacterial metabolic pathways in CFLs. Function prediction of the bacteria **(A)** and fungi **(B)** in CFLs. Volcano plot of the numbers of differentially expressed genes between the two groups **(C)**. Red represents the up-regulated, and green represents the down-regulated genes in group A. Blue represents that these genes had no differential expression between the two groups.

Function predictions derived from the microbial community data were obtained using PICRUSt for bacterial functional genes and FUNGuild for fungal functional genes to analyze microbial function (Figures 8A,B). The abundance of functional gene sequences in fungi was much lower than in bacteria. The functions of bacteria and fungi in CFLs include nucleotide biosynthesis, amino acid biosynthesis, fatty acid and lipid biosynthesis, and fermentation. The abundance of many bacterial metabolic pathways increased significantly after fermentation, whereas fungal metabolic pathways showed the opposite trend. For samples with *T. aurantialba* SCT-F3 fermentation medium (J0, J20, and J40), the abundance of functional genes increased, peaked on the 20th day of fermentation, and then gradually decreased. The relative abundance of functional bacterial genes at P40 increased significantly. There were 65 functional genes up-regulated and 11 down-regulated in samples inoculated with *T. aurantialba* SCT-F3 (J0, J20, and J40) compared with samples inoculated with potato glucose broth (P0, P20, and P40) (Table 2 and Figure 8C). The abundance of functional genes related to amino acid metabolism, fermentation, and fatty acid degradation increased after adding the microbial medium.

**Table 2:** Significant differential functional genes.

No	Metabolic pathway	Fold change	*p* values	Sign
1	enterobacterial common antigen biosynthesis	0.18	0.0108	Down
2	glycogen degradation II (eukaryotic)	0.51	0.0374	Down
3	polymyxin resistance	0.28	0.0411	Down
4	sulfoglycolysis	0.11	0.0207	Down
5	superpathway of (Kdo)2-lipid A biosynthesis	0.38	0.0414	Down
6	superpathway of demethylmenaquinol-6 biosynthesis I	0.64	0.0190	Down
7	superpathway of L-arginine and L-ornithine degradation	0.26	0.0212	Down
8	superpathway of lipopolysaccharide biosynthesis	0.20	0.0381	Down
9	superpathway of L-tryptophan biosynthesis	0.21	0.0087	Down
10	superpathway of ornithine degradation	0.22	0.0183	Down
11	thiazole biosynthesis II (Bacillus)	0.28	0.0197	Down
12	2-amino-3-carboxymuconate semialdehyde degradation to 2-oxopentenoate	15.45	0.0146	Up
13	2-aminophenol degradation	10.29	0.0126	Up
14	2-nitrobenzoate degradation I	13.67	0.0166	Up
15	3-phenylpropanoate degradation	2.82	0.0026	Up
16	4-coumarate degradation (anaerobic)	25.80	0.0320	Up
17	4-hydroxyphenylacetate degradation	1.88	0.0155	Up
18	adenosylcobalamin biosynthesis II (late cobalt incorporation)	2.64	0.0052	Up
19	aerobactin biosynthesis	174.72	0.0380	Up
20	aerobic respiration I (cytochrome c)	1.72	0.0057	Up
21	androstenedione degradation	5.22	0.0004	Up
22	catechol degradation II (meta-cleavage pathway)	4.58	0.0069	Up
23	chitin derivatives degradation	–	0.0134	Up
24	coenzyme B biosynthesis	13.13	0.0256	Up
25	coenzyme M biosynthesis I	37.84	0.0375	Up
26	D-cycloserine biosynthesis	4.52	0.0147	Up
27	ectoine biosynthesis	4.11	0.0010	Up
28	ergothioneine biosynthesis I (bacteria)	12.74	0.0004	Up
29	ethylmalonyl-CoA pathway	14.56	0.0048	Up
30	factor 420 biosynthesis	9.63	0.0054	Up
31	gallate degradation I	2.53	0.0107	Up
32	gallate degradation II	2.96	0.0061	Up
33	GDP-D-glycero-α-D-manno-heptose biosynthesis	6.40	0.0136	Up
34	glycerol degradation to butanol	5.02	0.0029	Up
35	glycine betaine degradation I	2.87	0.0319	Up
36	isopropanol biosynthesis	2.64	0.0347	Up
37	L-arabinose degradation IV	3.35	0.0008	Up
38	L-glutamate degradation VIII (to propanoate)	21.25	0.0140	Up
39	L-leucine degradation I	1.77	0.0071	Up
40	L-lysine fermentation to acetate and butanoate	18.54	0.0058	Up
41	L-methionine salvage cycle III	2.75	0.0109	Up
42	L-tryptophan degradation IX	9.11	0.0221	Up
43	L-tryptophan degradation to 2-amino-3-carboxymuconate semialdehyde	3.50	0.0336	Up
44	L-tryptophan degradation XII (Geobacillus)	9.47	0.0017	Up
45	L-tyrosine degradation I	1.63	0.0428	Up
46	mannan degradation	6.56	0.0429	Up
47	meta-cleavage pathway of aromatic compounds	3.28	0.0059	Up
48	methanol oxidation to carbon dioxide	8.13	0.0009	Up
49	methyl ketone biosynthesis	14.63	0.0173	Up
50	methylaspartate cycle	4.47	0.0212	Up
51	methylgallate degradation	2.91	0.0057	Up
52	mevalonate pathway II (archaea)	16.17	0.0414	Up
53	mycothiol biosynthesis	10.02	0.0172	Up
54	NAD biosynthesis II (from tryptophan)	3.39	0.0334	Up
55	nitrate reduction I (denitrification)	10.66	0.0416	Up
56	peptidoglycan biosynthesis V (β-lactam resistance)	9.54	0.0000	Up
57	phospholipases	12.84	0.0246	Up
58	photorespiration	2.97	0.0006	Up
59	protocatechuate degradation I (meta-cleavage pathway)	2.99	0.0057	Up
60	purine nucleobases degradation I (anaerobic)	3.54	0.0233	Up
61	pyrimidine deoxyribonucleotides biosynthesis from CTP	29.56	0.0373	Up
62	pyruvate fermentation to acetone	2.32	0.0488	Up
63	pyruvate fermentation to butanoate	3.15	0.0063	Up
64	pyruvate fermentation to propanoate I	4.20	0.0010	Up
65	reductive acetyl coenzyme A pathway	23.43	0.0022	Up
66	reductive TCA cycle II	–	0.0032	Up
67	superpathway of bacteriochlorophyll a biosynthesis	1.81	0.0437	Up
68	superpathway of C1 compounds oxidation to CO_2_	3.54	0.0489	Up
69	superpathway of *Clostridium acetobutylicum* acidogenic fermentation	3.12	0.0057	Up
70	superpathway of demethylmenaquinol-6 biosynthesis II	–	0.0341	Up
71	superpathway of menaquinol-8 biosynthesis II	9.88	0.0020	Up
72	superpathway of polyamine biosynthesis II	2.63	0.0208	Up
73	superpathway of pyrimidine ribonucleosides degradation	–	0.0398	Up
74	superpathway of UDP-N-acetylglucosamine-derived O-antigen building blocks biosynthesis	5.29	0.0037	Up
75	syringate degradation	2.93	0.0051	Up

“- “meant that functional genes were not detected in samples inoculated with potato glucose broth (P0, P20, and P40). Functional genes were up-regulated in samples inoculated with *T. aurantialba* SCT-F3 (J0, J20, and J40) compared to P0, P20, and P40. Functional genes were down-regulated in samples inoculated with fermented medium (J0, J20, and J40) compared to those inoculated with unfermented medium (P0, P20, and P40).

**Figure 9:**
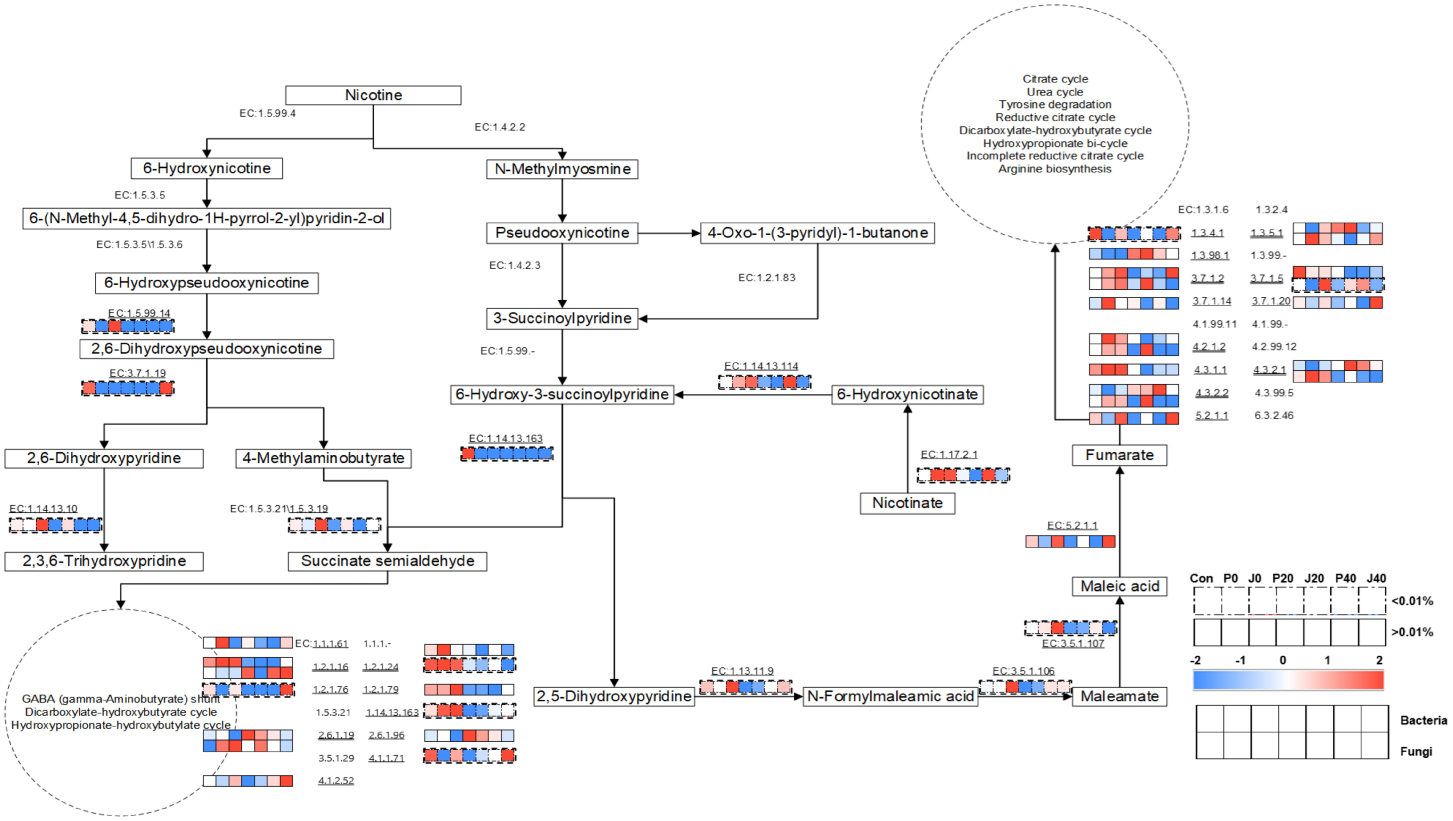
Metabolic pathways of nicotine degradation and nicotinate degradation and the expression levels of the genes encoding related enzymes in CFLs according to Encyclopedia of Genes and Genomes (KEGG) (Jimenez et al., 2008; Qiu et al., 2012).

Furthermore, a metabolic network, including nicotine degradation (M00810 and M00811) and nicotinate degradation (M00622) according to KEGG,^1^ during fermentation was constructed using the annotated enzymes and their metabolic pathways (Figure 9). The types and abundances of bacterial enzymes were higher than those of fungi. The enzymes associated with nicotine degradation were less abundant than those associated with nicotinate degradation. In the nicotine degradation pathway, EC:1.5.99.4 (nicotine dehydrogenase subunit A/B/C, ndhA/B/C), EC:1.4.2.2 (nicotine dehydrogenase, nox/nicA2), EC:1.5.3.5 ((S)-6-hydroxynicotine oxidase, nctB), EC:1.4.2.3 (pseudooxynicotine dehydrogenase, pao), EC:1.5.3.6 ((R)-6-hydroxynicotine oxidase, 6-hdno), EC:1.2.1.83 (3-succinoylsemialdehyde-pyridine dehydrogenase, sap), and EC:1.5.99 (3-succinoylpyridine monooxygenase) were not detected in any sample. The abundance of EC:1.5.99.14 (6-hydroxypseudooxynicotine dehydrogenase subunit alpha, kdhA), EC:1.14.13.10 (2,6-dihydroxypyridine 3-monooxygenase), and EC:1.5.3.19 (4-methylaminobutanoate oxidase (formaldehyde-forming)) increased in J0 and gradually decreased during fermentation. In the nicotinate degradation pathway, the abundance of EC:1.13.11.9 (2,5-dihydroxypyridine 5,6-dioxygenase, nicX), EC:3.5.1.106 (N-formylmaleamate deformylase, nicD), and EC:3.5.1.107 (maleamate amidohydrolase, nicF) showed the same trend. The abundance of microbial enzymes annotated to succinate semialdehyde transferred into the gamma-aminobutyrate (GABA) shunt, dicarboxylate-hydroxybutyrate cycle, and hydroxypropionate-hydroxybutylate cycle, and fumarate transferred into the citrate cycle, urea cycle, tyrosine degradation, reductive citrate cycle, dicarboxylate-hydroxybutyrate cycle, hydroxypropionate bicycle, incomplete reductive citrate cycle, and arginine biosynthesis were higher than the abundance of enzymes involved in nicotine degradation and nicotinate degradation pathways.

### 3.8. Correlation analysis of the predominant microbes and VFCs

**Figure 10:**
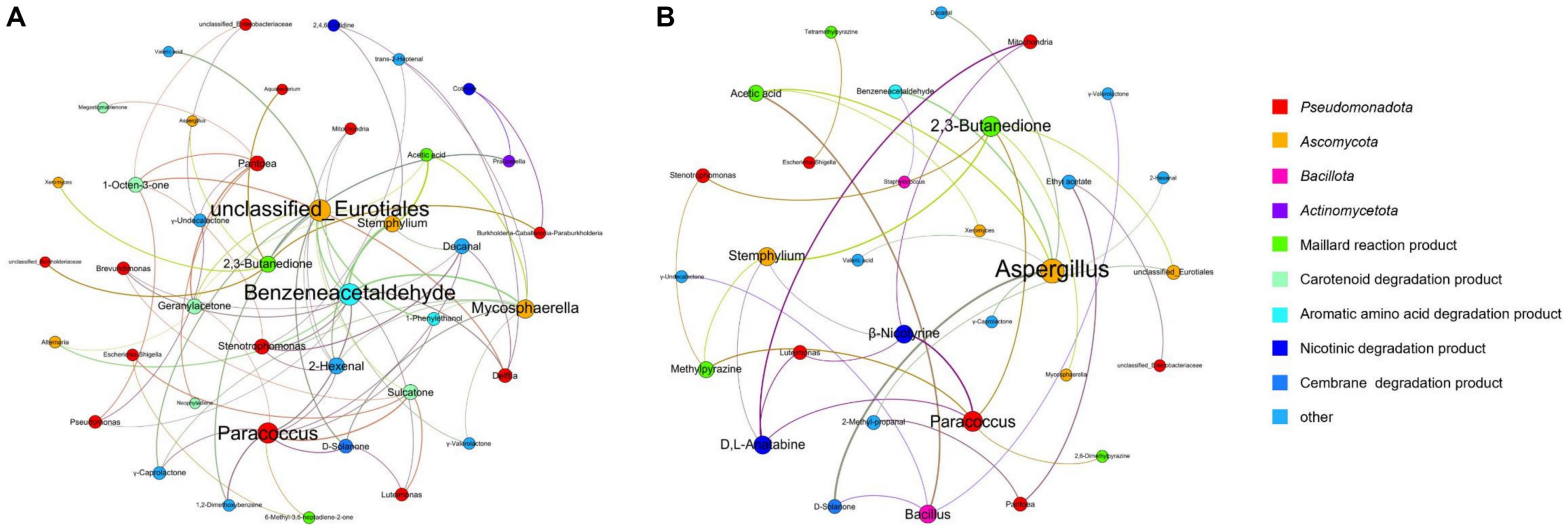
Co-occurrence networks of representative microbes and core VFCs in CFLs based on correlation analysis, positive **(A)** and negative **(B)** correlation.

Spearman’s correlation coefficients (*p < 0.05*) were used to establish significant associations between the 25 dominant microorganisms and 34 VFCs (Table 3). *Acinetobacter* (M1), *Aerococcus* (M2), and *Oceanobacillus* (M14) were not significantly correlated with VFCs. Moreover, 2-Ethyl-6-methylpyrazine, 2-phenylethanol, 4′-methylacetophenone, acetoin, acetophenone, and styrene were not significantly correlated with microorganisms. Graphs of the positive and negative-correlation networks were visualized using Gephi, as shown in Figures 10A,B, respectively. The modularity index of the positive correlation graph was 0.452 (>0.4), suggesting that the network had a modular structure. Twenty genera were significantly correlated with 21 VFCs values. A total of 41 notes and 72 edges (pairs of significant and robust correlations) were identified. There were six highly connected genera (degree ≥5), including *Pantoea*, *Paracoccus*, *Stenotrophomonas*, *Mycosphaerella*, *Stemphylium*, and unclassified *Eurotiales*, belonging to *Pseudomonadota* and *Ascomycota.* Seven highly connected VFCs, 1-octen-3-one, geranylacetone, sulcatone, 2,3-butanedione, benzeneacetaldehyde, 2-hexenal, and decanal, belong to plastochrome degradation products, Maillard reaction products, aromatic amino acid degradation products, and others. Nicotinic degradation products, cotinine, and 2,4,6-collidine were significantly and positively correlated with *Burkholderia-Caballeronia-Paraburkholderia* and *Prauserella*, *Mycosphaerella,* and *Stemphylium*, respectively. Gamma-valerolactone was significantly and positively correlated with *Mycosphaerella* and unclassified *Eurotiales*; geranylacetone with *Pantoea*; and unclassified *Eurotiales*, *Alternaria*, *Paracoccus*, and *Stemphylium*, neophytadiene, and megastigmatrienone with *Pantoea.*

**Table 3:** Spearman’s correlation coefficients between representative microbes and core volatile flavor compounds.

Spearman correlation coefficients	M3	M4	M5	M6	M7	M8	M9	M10	M11	M12	M13	M15	M16	M17	M18	M19	M20	M21	M22	M23	M24	M25
1,2-Dimethoxybenzene													0.60								0.61	
1-Octen-3-one							0.60					0.56			0.54			0.47		0.44		
1-Phenylethanol											0.46		0.53								0.67	
2,3-Butanedione		0.59	0.49			0.61					−0.48		−0.54	0.61			−0.60	−0.53	0.61		−0.48	0.56
2,4,6-Collidine											0.46						0.49					
2,6-Dimethylpyrazine													−0.47									
2-Hexenal			−0.44							0.45	0.48		0.51				0.62	0.51			0.48	
2-Methyl-propanal												−0.54									−0.48	
6-Methyl-3,5-heptadiene-2-one								0.44					0.46									
Acetic acid			−0.56	−0.66							0.57						0.69				0.44	−0.46
Benzeneacetaldehyde	0.60		−0.62		0.56		0.59		0.51	0.46	0.74		0.63		0.44	−0.47	0.80	0.58			0.67	
D,L-Anatabine									−0.50	−0.61			−0.50				−0.45					
Decanal			−0.53		0.44		0.51						0.51				0.50	0.58				
D-Solanone			−0.74	−0.48					0.50		0.59		0.55								0.62	
Ethyl acetate												−0.57								−0.48		
Geranylacetone	0.44											0.64	0.44				0.44				0.59	
Megastigmatrienone												0.46										
Methylpyrazine													−0.56				−0.49	−0.46				
Neophytadiene												0.54										
Sulcatone					0.47			0.58	0.58				0.65								0.48	
Tetramethylpyrazine								−0.49														
trans-2-Heptenal							0.50											0.44				
Valeric acid			−0.44																		0.66	
*β*-Nicotyrine									−0.47	−0.47			−0.63				−0.43					
*γ*-Caprolactone			−0.50								0.49		0.50								0.61	
*γ*-Undecalactone				−0.48								0.46			0.51					0.46		
*γ*-Valerolactone				−0.48							0.47										0.50	
Cotinine						0.49								0.49								

The coefficient is significant (*p < 0.05*). M1, *Acinetobacter*, M2, *Aerococcus*, M3, *Alternaria*, M4, *Aquabacterium*, M5, *Aspergillus,* M6, *Bacillus*, M7, *Brevundimonas*, M8, *Burkholderia-Caballeronia-Paraburkholderia*, M9, *Delftia*, M10, *Escherichia-Shigella*, M11, *Luteimonas*, M12, *Mitochondria*, M13, *Mycosphaerella*, M14, *Oceanobacillus*, M15, *Pantoea*, M16, *Paracoccus*, M17, *Prauserella*, M18, *Pseudomonas*, M19, *Staphylococcus*, M20, *Stemphylium*, M21, *Stenotrophomonas*, M22, unclassified *Burkholderiaceae*, M23, unclassified *Enterobacteriaceae*, M24, unclassified *Eurotiales*, M25, *Xeromyces*.

The modularity index of the negative correlation graph was 0.605 (>0.4), suggesting that the network had a modular structure. Fourteen genera significantly correlated with 17 VFCs. A total of 31 notes and 35 edges (pairs with significant and robust correlations) were identified. There were three highly connected notes (degree ≥5), including *Aspergillus*, *Paracoccus*, and 2,3-butanedione. *Aspergillus* was significantly and negatively correlated with D-solanone, acetic acid, 2-hexenal, benzeneacetaldehyde, valeric acid, *γ*-caprolactone, and decanal, which are cembrane degradation products and Maillard reaction products. 2,3-Butanedione significantly and negatively correlated with *Mycosphaerella*, *Stemphylium*, unclassified *Eurotiales*, *Paracoccus*, and *Stenotrophomonas,* belonging to *Ascomycota* and *Pseudomonadota*. The nicotinic degradation products, *β*-nicotyrine, and D,L-anatabine were significantly and negatively correlated with *Stemphylium*, *Paracoccus*, *Mitochondria*, and *Luteimonas*. Gamma-valerolactone was significantly and negatively correlated with *Bacillus*; ethyl acetate with *Pantoea* and unclassified *Enterobacteriaceae*; 2,6-dimethylpyrazine with *Paracoccus*; methylpyrazine with *Paracoccus*, *Stemphylium*, and *Stenotrophomonas*; and tetramethylpyrazine with *Escherichia-Shigella*.

## 4. Discussion

Tobacco leaves are the most widely planted nonfood crops worldwide. The quality of tobacco leaves is affected by the breed, climate, soil, and culture conditions. Due to the poor quality of cigar tobacco grown in China at present, developing a new measure to improve their quality would have important economic benefits for both tobacco farmers and manufacturers. In the present study, the addition of an edible medicinal fungus, *T. aurantialba* SCT-F3 fermented broth as the fermentation medium improved the quality of CFLs. According to the results of sensory evaluation, the mellow aroma, richness, maturity, and sweetness of fermented CFLs increased, irritation decreased, and the sense of balance improved. The addition of fermentation media significantly changed the total alkaloid, total nitrogen, reduced sugar, and total sugar contents. The CFLs’ total sugar and reduced sugar contents significantly increased at the beginning of fermentation. The fermented broth of *T. aurantialba* contains not only a large amount of monosaccharides, such as mannose, glucose, and galactose (Deng et al., 2017), but also amylase and cellulase, which can decompose starch and cellulose (Chen et al., 2021), and might be the cause of this phenomenon. At the end of fermentation, there were no significant difference in the reducing sugar and total sugar content of all samples, and there was no significant difference in the total alkaloid content of the samples with added culture medium. Microbes consume nicotine and aniline compounds, and alkaloid compounds produced by microbial metabolism or protein decomposition, which may cause the total alkaloid content first decreased and then increased during fermentation. The fluctuation of total sugar and reducing sugar content during the fermentation process is due to the degradation of glycogen, the utilization of microorganisms, and the Maillard reaction (Ye et al., 2019). Total nitrogen and nicotine are related to smoke concentration and pungency, and sugar is related to sweetness (Hu et al., 2022). However, within a certain concentration range, the contents of chemical components, total alkaloids, total nitrogen, reducing sugar, and total sugar may not directly reflect the sensory qualities of CFLs. Therefore, we measured VFCs to explore the effects of *T. aurantialba* SCT-F3 fermentation medium on the quality of CFLs.

Aromatic amino acid degradation products, plastochrome degradation products, cembrane degradation products, Maillard reaction products, and nicotine degradation products are especially important for forming characteristic tobacco flavors and aromas (Xie, 2009; Yin et al., 2012). Four VFCs from the Maillard reaction, 2,3-butanedione with creamy fragrance, methylpyrazine with sweet fragrance, 2,6-dimethylpyrazine and 2-ethyl-6-methylpyrazine with baked potato fragrance (Yan and Zhao, 2008) increased in CFLs fermented with *T. aurantialba* SCT-F3 fermentation medium. Fermentation led to the increase in nicotine degradation products, including *β*-nicotyrine, D,L-anatabine, and cotinine. VFCs from *T. aurantialba* SCT-F3 broth increased in unfermented CFLs added *T. aurantialba* SCT-F3 fermentation medium. However, the increase in VFCs did not significantly improve the sensory quality scores. Some VFCs in CFLs gradually decreased or increased as fermentation progressed; however, the sensory quality continuously increased. Additionally, through PLS analysis of conventional chemical constituents, VFCs, and sensory evaluations, it was found that in the complex aroma system by our study, 2-ethyl-6-methylpyrazine, methylpyrazine, D,L-anatabine, *β*-nicotyrine, nicotinic degradation products and total nitrogen had a significantly positive contribution to the sensory quality. In contrast, 1-phenylethanol with rose flavor, geranylacetone with green flavor, megastigmatrienone with flowery and woody flavor, D-solanone enhancing the fragrance of tobacco, 6-methyl-3,5-heptadiene-2-one with cinnamon aroma, 1,2-dimethoxybenzene with pleasant odor, and *γ*-caprolactone with herbal and sweet flavor showed the opposite trend. Although higher content of VFCs would endow CFLs with more fragrant flavor, the coordination and balance of VFCs in tobacco leaves determine the sensory quality of tobacco leaves. A significant increase or decrease in a VFC might break the balance (Zhang et al., 2021). However, there is no criteria for evaluating coordination and balance to measure the quality of cigar tobacco yet. Further study is needed to establish the criteria for evaluating coordination and balance of chemical composition and VFCs to reduce sensory evaluators workload and provide objective criterion.

The effects of *T. aurantialba* SCT-F3 fermentation medium and fermentation on the microbial community of CFLs were amplified using Illumina high-throughput sequencing. The addition of the fermentation medium significantly changed the microbes’ richness, diversity, and structure. Fungal communities have more stable structures than bacterial communities. *Aspergillus* was the dominant fungus, mainly clustered in CFLs, and may play an important role in maintaining the stability of fungal communities. *Aspergillus* species, filamentous fungi with GRAS status, are widely used in the food industry to produce multiple enzymes such as amylases, proteases, lipases, cellulases, and aroma compounds (Raveendran et al., 2018; Kumar et al., 2021; Chilakamarry et al., 2022). It is worth noting that in the fermentation process of CFLs, avoiding *Aspergillus* sp. mycelium formation affects the appearance quality of CFLs, while applying their properties of enzyme and aroma production is very important.

Changes in the microbial communities of the CFLs may be separated into three distinct phases. Phase I is the initial disturbance period. In this phase, the endogenous microbial community and its functions changed dramatically with the addition of fermentation media. Nutrients, including sugars, proteins, ketones, aldehydes, alcohols, and esters from *T. aurantialba* SCT-F3 fermentation medium facilitated the growth of 31 microbial genera, which led to an increase in diversity and species richness. Furthermore, the activity of 20 microbial functional genes increased, including nucleotide biosynthesis, amino acid biosynthesis, fatty acid and lipid biosynthesis, fermentation, nicotine degradation, and nicotinate degradation. This indicates that adding *T. aurantialba* SCT-F3 accelerated the VFCs of CFLs and shortened the fermentation time (Cai et al., 2022). Phase II was the transitional period. The loss of diversity and species richness likely resulted from the interactions between microorganisms, such as competition and antagonism (Tao et al., 2014). Functional genes related to nucleotide biosynthesis, amino acid biosynthesis, fatty acid, and lipid biosynthesis, and fermentation further increased, indicating that substance consumption and new substance synthesis occurred in CFLs during this period. Phase III was relatively stable. The microbial community gradually stabilized owing to the complete suppression of external disturbances (Wu et al., 2016). The abundance of functional genes was low, indicating that the metabolic activity of microorganisms has also become less active.

VFCs are affected by microbial metabolism (Yang et al., 2021). Network analysis has shown that microbes in CFLs are closely related to VFCs, consistent with previous study findings (Zhang et al., 2020a,b; Zheng et al., 2022a,b). *Aspergillus* sp. produces aromatic compounds (Chilakamarry et al., 2022); this was positively related to 2,3-butanedione in our study. *Alternaria*, a cosmopolitan fungal genus, is one of the dominant resident fungi in tobacco leaves (Chattopadhyay et al., 2021) and is positively related to benzene acetaldehyde with a fruity and sweet aroma (Koan Sik et al., 2006), and geranylacetone. *Pseudomona*s was positively related to 1-octen-3-one with a mushroom flavor (Bauer et al., 2022), *γ*-undecalactone with a sweet peach flavor (Chen et al., 2022), and benzeneacetaldehyde with a hyacinth aroma. *Achromobacter*, *Cellulomonas*, *Enterobacter*, *Arthrobacter*, *Alcaligene s*, *Pseudomonas*, and *Aspergillus* have been reported to degrade nicotine (Meng et al., 2010). In our study, *Burkholderia-Caballeronia-Paraburkholderia* and *Prauserella* were positively correlated with cotinine and *Mycosphaerella* and *Stemphylium* were positively correlated with 2,4,6-collidine. Although microbial enzymes that catabolize nicotine to 6-hydroxynicotine (EC:1.5.99.4) or N-methylmyosmine (EC: 1.4.2.2) were not detected, enzymes associated with nicotine degradation and the nicotinate degradation pathway increased after the addition of *T. aurantialba* SCT-F3 fermentation medium. Microbial enzymes mainly participate in succinate semi-aldehyde-transfer and fumarate-transfer pathways. Hence, it is speculated that microbial enzymes in CFLs catalyze the increase of *β*-nicotyrine, D,L-anatabine, and cotinine during fermentation; some intermediates of these four strains participate in the transformation process of nitrogen-containing substances and some strains use nitrogen-containing substances in the metabolic process.

## 5. Conclusion

When spontaneous fermentation cannot meet the demands of consumers, medium fermentation is an important processing method for improving the sensory quality of cigar tobacco leaves. Our present study demonstrates a new microbial medium fermentation method for improving the quality of CFLs. The addition of the microbial medium produced by *Tremella aurantialba* SCT-F3 significantly affected the chemical components and VFCs, mainly increasing the content of Maillard reaction products and nicotine degradation products. The fermentation medium changed the microbial community’s structure and improved CFL microbial activity. We also revealed the contributions of chemical components and VFCs to sensory evaluation and the association between predominant microbes and VFCs.

## Data availability statement

The data presented in the study are deposited in the NCBI BioProject repository, accession number PRJNA999475.

## Author contributions

QiZ: Conceptualization, Data curation, Formal analysis, Methodology, Software, Writing – original draft, Writing – review & editing. SY: Investigation, Methodology, Resources, Writing – review & editing. ZY: Investigation, Methodology, Resources, Writing – review & editing. TZ: Investigation, Methodology, Resources, Writing – review & editing. PL: Investigation, Methodology, Resources, Writing – review & editing. QuZ: Investigation, Methodology, Resources, Writing – review & editing. WC: Investigation, Methodology, Resources, Writing – review & editing. YW: Investigation, Methodology, Resources, Writing – review & editing. JZ: Funding acquisition, Supervision, Writing – review & editing. XJ: Investigation, Methodology, Resources, Writing – review & editing. DL: Resources, Supervision, Writing – review & editing.

## Footnotes

^1^ https://www.kegg.jp/

## Abbreviations

CFL: Cigar filler leaf
OTU: Operational taxonomic unit
PLS: Partial least-squares
VFC: Volatile flavor compound
