# Supplementary material for: Effects of a novel microbial fermentation medium produced by Tremella aurantialba SCT-F3 on cigar filler leaf
Source: Front Microbiol. 2023 Sep 22;14:1267916. doi: 10.3389/fmicb.2023.1267916 (PMC10556473; doi:10.3389/fmicb.2023.1267916)
Supplement: Supplementary file 1 [file Table_1.DOCX]

**Supplementary Table 1** Contents of volatile flavor compounds of unfermented potato glucose broth and *T. aurantialba* SCT-F3 fermented potato glucose broth (mg/L)

| Name | unfermented potato glucose broth | *T. aurantialba* SCT-F3 fermented potato glucose broth |
| --- | --- | --- |
| Ethanol | 2.75±0.26 | 30.47±0.41 |
| Isobutyraldehyde | 0.02±0.00 | - |
| Ethyl acetate | 0.04±0.02 | 0.34±0.01 |
| Isobutanol | - | 5.90±0.25 |
| 3-Methylbutanal | 0.43±0.05 | 0.06±0.01 |
| 2-Methylbutanal | 0.11±0.03 | 0.01±0.00 |
| Methyl isobutyrate | - | 0.04±0.01 |
| 2,3-Pentanedione | 0.11±0.01 | - |
| Isoprenol | - | 0.02±0.00 |
| Isoamylol | - | 15.79±0.42 |
| Pyrazine | 0.12±0.02 | - |
| 2-methylbutan-1-ol | - | 11.14±1.20 |
| Acetoin | 0.01±0.01 | 0.01±0.01 |
| Pyridine | 0.05±0.01 | - |
| Pyrrole | 0.03±0.02 | - |
| Isobutyric acid | - | 0.05±0.04 |
| Methyl isovalerate | - | 0.29±0.13 |
| 3-Methylbut-2-enal | - | 0.06±0.01 |
| 2-Methyloxolan-3-one | 0.02±0.00 | - |
| 2-Methylpyrazine | 0.10±0.00 | 0.02±0.00 |
| Furfural | 0.15±0.01 | 0.35±0.01 |
| Methyl 3,3-Dimethylacrylate | - | 0.09±0.03 |
| 2-Octanone | 1.07±0.41 | 0.44±0.24 |
| Methyl tiglate | - | 0.07±0.02 |
| 1-Hexanol | 0.03±0.02 | 0.07±0.02 |
| *p*-Xylene | 0.05±0.00 | 0.07±0.02 |
| 3-Heptanone | 0.05±0.04 | - |
| 2-Heptanone | 0.12±0.04 | - |
| Styrene | 0.09±0.05 | 0.04±0.02 |
| Methional | 0.04±0.01 | - |
| 2-Acetylfuran | 0.01±0.00 | - |
| 2,5-Dimethyl pyrazine | 0.84±0.02 | 0.26±0.01 |
| Methyl furan-3-carboxylate | - | 0.56±0.04 |
| 4-Methylheptan-2-one | 0.16±0.01 | 0.03±0.02 |
| (E)- 2-Heptenal | 0.06±0.01 | - |
| Heptan-1-ol | 0.36±0.03 | 0.23±0.04 |
| 2-Acetyl-5-methylfuran | - | 0.05±0.00 |
| Dimethyl trisulfide | 0.03±0.00 | - |
| 7-Octen-4-ol | 0.41±0.02 | 0.87±0.37 |
| 3-Octanone | - | 0.13±0.13 |
| 3-Octanol | - | 0.08±0.04 |
| 2-Acetylthiazole | 0.14±0.00 | - |
| 2-Ethylhexanol | 0.21±0.02 | 0.66±0.33 |
| Limonene | 0.01±0.01 | 0.04±0.04 |
| Phenylacetaldehyde | 0.28±0.01 | 0.13±0.01 |
| 2-Nonanone | 0.01±0.00 | - |
| Dodecane | 0.08±0.02 | 1.31±1.26 |
| 2-Methyl-5-(1-methylethyl)-pyrazine | 0.01±0.01 | 0.01±0.01 |
| Acetophenone | 0.06±0.00 | 0.05±0.01 |
| 3,5-octadiene-2-one | 0.01±0.00 | - |
| 1-Octanol | 0.10±0.00 | 0.05±0.04 |
| Benzaldehyde, 2-methyl- | 0.34±0.25 | 0.69±0.25 |
| Pyrazine, 3-ethyl-2,5-dimethyl- | 0.06±0.01 | 0.03±0.00 |
| (E,E)- 3,5-Octadien-2-one, | 0.06±0.00 | - |
| Methyl benzoate | - | 42.83±9.00 |
| Nonanal | 0.05±0.01 | 0.31±0.18 |
| Benzeneethanol | 0.01±0.01 | 0.24±0.03 |
| 1-Methyl-4-(1-methylethyl)-benzene | - | 0.01±0.01 |
| 1,2,4,5-Tetramethyl-Benzene | 0.16±0.14 | 0.05±0.04 |
| Ethyl benzoate | - | 2.25±1.50 |
| L-Menthol | 0.05±0.00 | 0.27±0.13 |
| 2-Methyl-Benzofuran | - | 0.07±0.01 |
| Naphthalene | 0.04±0.01 | 0.03±0.01 |
| Methyl salicylate | 0.19±0.01 | 0.20±0.10 |
| Methyl 4-methylbenzoate | - | 0.43±0.24 |
| 2,4-Dimethylbenzaldehyde | 0.17±0.01 | 0.05±0.00 |
| Citronellol | - | 0.13±0.05 |
| 3-Phenyl-furan | 0.01±0.00 | - |
| Benzothiazole | 0.05±0.00 | 0.14±0.02 |
| 3-Phenylpropionic acid methyl ester | - | 0.02±0.01 |
| 1H-Indole | 0.02±0.01 | - |
| 4'-Isopropylacetophenone | 0.05±0.01 | - |
| γ-Heptalactone | 0.01±0.00 | - |
| Propane acid 2-methyl-3-hydroxy-2,4,4-trimethylpentyl ester | 0.03±0.01 | - |
| Methyl cinnamate | 0.05±0.00 | 2.23±0.68 |
| Ethyl p-Anisate | - | 0.57±0.14 |
| 1-Dodecanol | 0.05±0.00 | 0.09±0.08 |
| β-Eudesmol | - | 0.23±0.20 |
| Nerolidol | - | 0.49±0.32 |
| Cedrol | 0.02±0.01 | - |
| Globulol | - | 0.50±0.18 |
| 2-Propenoic acid, 3-(4-methoxyphenyl)-, methyl ester | - | 0.07±0.02 |
| Benzene, 1,1'-(1,1,2,2-tetramethyl-1,2-ethanediyl)bis- | 0.01±0.00 | - |
| Phytone | 0.01±0.00 | - |
| Hexadecanoic acid, ethyl ester | - | 0.31±0.13 |

Note: - meant not detected.
